# Exercise therapy, quality of life, and activities of daily living in patients with Parkinson disease: a small scale quasi-randomised trial

**DOI:** 10.1186/1745-6215-10-67

**Published:** 2009-08-11

**Authors:** Bahram Yousefi, Vahid Tadibi, Ali Fathollahzadeh Khoei, Ali Montazeri

**Affiliations:** 1 Faculty of Physical Education, Razi University, Kermanshah, Iran; 2 Iranian Institute for Health Sciences Research, ACECR, Tehran, Iran

## Abstract

**Background:**

The purpose of this study was to examine the effect of a 10-week exercise therapy regimen on activities of daily living (ADL) and perceived health status in patients with Parkinson disease.

**Methods:**

Twenty-four Parkinson's disease patients entered into the study. Participants were allocated into the experimental (n = 12) or control group (n = 12). ADL was assessed using the Short Parkinson Evaluation Scale/Scale for Outcomes in Parkinson Disease (SPES/SCOPA) and perceived health status was measured using the Parkinson's Disease Quality of Life (PDQL) questionnaire. Patients in the experimental group received pharmacological therapy plus a 1-hour exercise therapy session 4 times a week, while patients in the control group received pharmacological therapy only. The Mann-Whitney U test was used for comparison.

**Results:**

The mean age of participants was 59.8 (SD = 3.0) and 58.2 (SD = 3.4) years in the experimental and control groups, respectively. The median Hoehn and Yahr stage was 3.0 for both groups. There were no significant differences in all subscales and overall scores between two groups at baseline. However, after the intervention, except for the emotional functioning (P = 0.27), there were significant differences between the two groups for Parkinson symptoms, systemic symptoms, social functioning, and overall scores of the PDQL (all P values < 0.05), and the ADL (P = 0.01) indicating that quality of life was improved in the experimental group.

**Conclusion:**

The findings from this small scale quasi-randomised trial showed that exercise therapy was effective in improving activities of daily living and perceived health status in patients with Parkinson's disease. Indeed, exercise therapy could be offered to patients with Parkinson disease, considering that it is low in cost and usually has no negative side effects.

**Trial registration:**

Current Controlled Trials ISRCTN98825027

## Background

Parkinson disease is a complex neurodegenerative condition with both motor and non-motor symptoms [1]. Due to a progressive loss of substantia nigra neurons, which produce dopamine, neurotransmitter imbalances occur in the basal ganglia [2]. If around 80% of neurons have been lost, Parkinson disease become evident and patients begin to experience a vide variety of difficulties [3].

The most clinical features of Parkinson disease are motor symptoms including tremor at rest, rigidity, and bradykinesia [4]. These impairments cause decline in functional status so that the patients cannot cope with tasks such as walking, rising from a chair, moving in bed, eating, or putting on shoes [5]. Limitation in functional status and activities of daily living often results in a loss of independence and decline in quality of life. It has been shown that patients with Parkinson disease, compared to the general population, have lower mobility and physical functioning [5,6]. Therefore, it can be expected that, as a result of the combined Parkinson disease impairment and inactivity, patients with Parkinson disease could enter into a downward spiral of immobility; which causes progressively increasing deficits in muscle strength, and quality of life [5].

Management of patients with Parkinson disease should be a multidisciplinary approach, which includes a coordination of pharmacological and non-pharmacological treatment [1,7]. It has been suggested that physical therapy as an effective non-pharmacological treatment has positive effects on mobility and activities of daily living in individuals with Parkinson disease [8]. Physical therapy in conjunction with routine medication for patients with Parkinson disease could break the downward spiral of immobility. However, only few randomised trials have investigated the effects of physical therapy on the quality of life of patients with Parkinson disease [9-14]. Although a recent meta-analysis of fourteen randomised trials of the effectiveness of exercise interventions supported that exercise was beneficial in improving physical functioning and health-related quality of life in patients with Parkinson disease, it indicated that questions about optimal content of exercise interventions (dosing and components) remain to be answered [15].

The aim of this small scale study was to investigate whether an exercise therapy regimen is effective in improving activities of daily living and quality of life in patients with Parkinson disease in order to add this non-pharmacological intervention as a routine regimen for suitable candidate among our patients in Iran.

## Methods

### Ethics

The ethics committee of Razi University (Kermanshah, Iran) approved the study. All patients gave their consent.

### Participants

A consecutive sample of 24 male patients with an idiopathic Parkinson disease diagnosed by a neurologist, with the following criteria were entered into the study: age ≤ 65 years, stage 2 to 3 of the Hoehn and Yahr scales [16], no cardiovascular, orthopaedic, or other neurological disease, no cognitive impairment (MMSE score >24; [17]), and had not received exercise therapy within the 6 months prior to the study.

### Procedure

Before the beginning of the intervention, every other patient was allocated into the control or experimental group (12 participants in each group) by a nurse who was not connected to the study. She was responsible to explain the study and randomly allocate patients to the study arms and ask their permission for the trial. During the study period, all patients in both groups continued their routine pharmacological treatment. The pharmacological treatment for all patients was the same and remained unchanged during the study period. Patients received Sinmet (Levodopa-c 100 mg/10 mg, twice per day), Artan (Trihexyphinidyl, 1 mg per day), Selegiline (5 mg twice per ay) and Amantadin (100 mg twice per day) as their medication. Both groups completed activities of daily living and quality of life measures twice: once before intervention and once one month after exercise sessions and group meetings. A physiotherapist who was blind to the group allocation and was not connected to the study supervised the procedure and helped to collect the data. There were no withdrawals.

### Intervention

Exercise therapy consisted of 10 weeks in which participants in the experimental group, in conjunction with their pharmacological therapy, came to the clinic 4 days a week about 1 hour after taking their PD medications. Each exercise therapy session lasted for 1 hour, beginning with about 10 minutes warm-up (walking, moderate stretching and breathing training). Main body of the session lasted 40 to 50 minutes and included three parts: postural, stretching, and strengthening exercises recommended for PD patients by the Parkinson Society Canada. The reason for choosing this programme was due to its availability, simplicity, and instructive pictures. For each part there were several practical training and movements such as standing, sitting, waking up, a range of movement for joints and muscles by holding positions and relaxing, and strengthening body muscles exercises [see ]. Each session was ended with 5 minutes of relaxation and breathing training. One of the authors led the patients through the same controlled standardized series of exercises at each session. Participants in the control group received only their pharmacological therapy. However, they also gathered together 4 times a week to receive general lectures on health topics and PD, attention and the feeling of being in a group. The authors also controlled these sessions. There were 6 participants during each session of the exercise and control group interventions.

### Measures

The activities of daily living (ADL) subscale of the Short Parkinson Evaluation Scale/Scales for Outcomes in Parkinson disease (SPES/SCOPA) was used to assess activities of daily living of the participants. Marinus et al. developed the scale and they reported that it is a reliable and valid measure and can be used both in research settings and clinical practices [18]. The ADL subscale of the SPES/SCOPA consists of 7 questions about speech, feeding, dressing, hygiene, changing position, walking, and hand writing with 4 response categories (from normal = 0 to severe = 3). This gives scores ranging from 0 to 21 with higher scores indicating a poorer condition.

Perceived health status was measured using the Parkinson Disease Quality of Life (PDQL) questionnaire. The PDQL is a self-administered measure which contains 4 subscales: Parkinson symptoms (PS- 14 items), systemic symptoms (SS- 7 items), social functioning (SF- 7 items), and emotional functioning (EF- 9 items); and an overall score can be derived, with a higher score indicating better perceived quality of life [19]. Each item is rated on a 5-point scale. This gives scores ranging from 0 to 70, 0 to 35, 0 to 35, 0 to 45, and 0 to 185 for PS, SS, SF, EM and total scores respectively.

Both instruments were translated from English to Persian (the Iranian language) using forward-backward translation method [20]. Internal consistency for the ADL and the PDQL questionnaires were measured and showed satisfactory results (Cronbach's α equal or greater than 0.70).

### Statistical analysis

The Mann-Whitney U test was used to compare the patients' characteristics, activities of daily living, and quality of life scores between the two groups at baseline (pre-test) and at follow-up (post-test). Also similar analysis was performed to compare changes in scores (post-test minus pre-test scores) between the experimental and the control groups. All statistical analyses were performed using SPSS for windows, version 15.0 and statistical significance was set at p < 0.05.

## Results

In all 24 male patients were entered into the study. During patient allocation there were 8 female and 4 male patients that screened for eligibility but failed. None of the patients started the study and were allocated to experimental or control groups dropped out. The mean age of participants was 59.8 (SD = 3.0) and 58.2 (SD = 3.4) years in the experimental and control groups, respectively. The median Hoehn and Yahr stage was 3.0 for both groups. There were no significant differences between the two groups with regard to their demographic and clinical status (Table 1).

**Table 1:** The characteristics of experimental and control groups

	**Experimental group (n = 12)**	**Control group (n = 12)**	
	**Mean (SD)**	**Mean (SD)**	**P**

**Age (year)**	59.8 (3.0)	58.2 (3.4)	0.24

Range	54–65	52–64	

**Disease duration (year)**	4.8 (1.1)	4.4 (1.2)	0.39

**Stage (Hoehn and Yahr scale)**	2.8 (0.3)	2.7 (0.4)	0.58

Median	3.0	3.0	

1^st ^quartile	3.0	2.2	

Range	2–3	2–3	

The main findings of the study are summarized in Tables 2 and 3. There were no significant differences in all subscales and overall scores of the PDQL as well as the ADL score between the two groups at baseline. However, after treatment, except for the emotional functioning, there were significant differences between the two groups indicating that quality of life and activities of daily living were improved in the experimental group.

**Table 2:** Comparison of pre-test PDQL and ADL scores between experimental and control groups

	**Experimental group (n = 12)**		**Control group (n = 12)**		
	**Mean (SD)**	**Median (range)**	**Mean (SD)**	**Median (range)**	**P**

PDQL score*					

PS	41.2 (9.8)	41.0 (24–60)	40.0 (9.7)	39.5 (25–58)	0.75

SS	17.8 (3.7)	18.0 (10–24)	17.6 (3.2)	17.5 (10–22)	0.83

EF	23.3 (6.4)	23.0 (12–40)	23.3 (4.1)	24.0 (14–30)	0.62

SF	18.1 (2.7)	18.5 (12–22)	17.6 (2.8)	18.5 (12–21)	0.72

Total score	100.7 (22.1)	101.0 (58–149)	98.6 (17.4)	103.0 (61–122)	0.88

ADL score**					

Activities of daily living score	7.0 (1.3)	7.0 (5–9)	6.4 (1.8)	7.0 (4–9)	0.44

Abbreviations. PDQL: the Parkinson Disease Quality of Life questionnaire; ADL: Activities of daily living; PS: Parkinson symptoms; SS: Systemic symptoms; EF: Emotional functioning; SF: Social functioning;

* Higher scores indicate better conditions.

** Higher score indicates a poorer condition.

**Table 3:** Comparison of post-test PDQL and ADL scores between experimental and control groups

	**Experimental group (n = 12)**		**Control group (n = 12)**		
	**Mean (SD)**	**Median (range)**	**Mean (SD)**	**Median (range)**	**P**

PDQL score*					

PS	48.3 (9.8)	51.0 (24–62)	38.7 (8.4)	39.5 (27–54)	0.01

SS	21.3 (4.3)	22.0 (10–26)	16.5 (3.0)	17.5 (10–20)	0.001

EF	21.1 (3.4)	22.0 (12–25)	21.5 (3.2)	22.5 (14–25)	0.54

SF	21.0 (3.3)	22.0 (12–25)	17.9 (3.2)	19.0 (12–22)	0.007

Total score	115.4 (22.3)	120.5 (56–140)	95.6 (15.6)	100.0 (65–116)	0.006

ADL score**					

Activities of daily living score	5.1 (0.7)	5.0 (4–6)	6.7 (1.5)	7.0 (4–9)	0.008

Abbreviations. PDQL: the Parkinson Disease Quality of Life questionnaire; ADL: Activities of daily living; PS: Parkinson symptoms; SS: Systemic symptoms; EF: Emotional functioning; SF: Social functioning;

* Higher scores indicate better conditions.

** Higher score indicates a poorer condition.

Finally the mean change scores between the experimental and control groups were compared. There were significant differences in score changes for all measures between experimental and the control groups (from pre to post-test) except for emotional functioning. The results are shown in Table 4.

**Table 4:** Comparison of mean changes between experimental and control groups

	**Experimental group** **(n = 12)**	**Control group** **(n = 12)**	
	**Mean change (SD)** **(post-test minus pre-test)**	**Mean change (SD)** **(post-test minus pre-test)**	**P**

PDQL*			

Parkinson symptoms	7.1 (4.9)	-1.3 (3.7)	0.001

Systemic symptoms	3.5 (2.3)	-1.1 (3.9)	0.001

Emotional functioning	-2.3 (5.2)	-1.8 (1.5)	0.27

Social functioning	3.0 (2.6)	0.3 (1.7)	0.004

Total PDQL	14.7 (11.3)	-3.0 (4.5)	0.001

ADL**			

Activities of daily living score	-1.9 (1.2)	0.3 (2.4)	0.01

* Negative values show deterioration.

** Negative value shows improvement.

## Discussion

As suggested Parkinson disease is in an active state of evolution [21]. The aim of this study was to determine whether exercise therapy would enhance activities of daily living and quality of life in patients with Parkinson disease. The results indicated that a 10-week exercise therapy regimen (a 1-hour session four times per week), in conjunction with the pharmacological therapy, could have positive effects on ADL and QOL in patients with Parkinson disease. These results are in accordance with other research findings where many investigators showed that for treatment of patients with Parkinson disease, combination of pharmacological therapy and exercise is more effective than using only the pharmacological therapy.

However, one should note that different rehabilitation programmes might show different effects. For instance, studies have shown that physical training could improve the QOL and some ADL components such as standing from chair, turning in bed, and getting in and out of bed [9], or an 8-week Pole-Striding exercise could increase perceived functional independence and QOL [10], or a 36-session of aerobic conditioning and strengthening training could significantly improve QOL [11]. In contrast, Burini et al. observed no effects for 40 aerobic and Qigong training sessions on the quality of life of Parkinson disease participants [12]. Keus et al. showed that physical therapy in conjunction with pharmacological treatment improved quality of life, but suggested that physical therapy is unlikely to influence the disease itself. However, they argued that physical therapy could improve daily functioning by teaching and training patients in the use of movement strategies [22]. In addition, a recent review of the literature indicated that there was insufficient evidence to support that exercise would reduce falls or depression in patients with Parkinson disease [15].

The study findings indicated a positive effect of exercise therapy regimen on social functioning in patients. In the PDQL questionnaire, social functioning includes abilities to perform social activities like hobbies, leisure activities, transport, and go on holiday [19]. These activities are linked to motor ability. Thus, the observed improvement might be explained by the fact that exercise therapy improved motor function that in turn improved social functioning in patients.

Emotional functioning was decreased in both groups. However, the deterioration was greater in the intervention group. This subscale is characterized with some feelings such as insecure feeling due to physical limitation, feeling embarrassed about disease, being afraid of progression of illness, depression, and concentration difficulties [19]. Decrease in scores for both groups might be attributed to patients' knowledge of disease progression. Nevertheless, as suggested the results highlight the need for providing psychological interventions in these patients in order to enhance QOL [23]. Studies have shown that even mood symptoms could be regarded as one of the main determinants of poor quality of life in patients with Parkinson disease [24].

The positive effects of physical training interventions on improvement of motor performance related to changes in position, which affects ADL, could be explained by the fact that physical activities could help to break the cycle of immobility [9]. In addition, stretching exercises could be helpful for patients with PD by maintaining and improving the range of motion, and therefore keeping muscle flexibility and strength [25]. Also stretching exercises could help PD patients to improve their posture due to enhancement of trunk mobility and activating of the extensor muscles [26]. Scandalis et al. reported that patients with PD, like other elderly people, could increase their muscle strength through a resistance-training program, and as a result, can enhance their posture, stride length, and gait speed [27].

We used a well-known questionnaire (the PDQL) to measure perceived health status in patients with PD. Studies of patients with Parkinson disease have shown that the psychometric properties of the questionnaire were desirable [28]. It is argued that using generic measures, such as the Short Form Health Survey (SF-36), to assess quality of life in patients with Parkinson disease might end with misleading results. For instance a study showed that the two SF-36 summary measures were not found to be valid indicators of physical and mental health in patients with Parkinson disease [29]. Indeed future studies of quality of life in patients with Parkinson disease should define what constitute health-related quality of life in PD patients at first place, and secondly employ sound measures to avoid misleading findings [30]. However, as suggested by Hagell and Nygren even in using such measures (e.g. the 39-item Parkinson's Disease Questionnaire-PDQ-39) endpoints should be interpreted cautiously particularly small but clinically important effects among patients with severe problems [31].

The very small sample size could be regarded as the main limitation of this study. In addition, because none of the female patients met the study inclusion criteria, women patients were not included in this study. This fact limits the external validity (generalizability) of the results. Also the short duration of the study and the fact that there was no follow-up to evaluate whether the differences in two groups maintained is another limitation. Finally, since patients were not blind there might be an overestimation of the intervention effect adherent to the study results. In fact, the study design was quasi-randomisation and thus failed to maintain adequate allocation concealment [32,33]

## Conclusion

Exercise therapy in conjunction with pharmacological therapy was effective in improving activities of daily living and perceived health status in patients with Parkinson disease. Indeed, exercise therapy could be offered to patients with Parkinson disease, considering that it is low in cost and usually has no negative side effects.

## Abbreviations

PD: Parkinson disease; PDQL: the Parkinson Disease Quality of Life questionnaire; ADL: Activities of daily living; QOL: Quality of life; SPES/SCOPA: the Short Parkinson Evaluation Scale/Scales for Outcomes in Parkinson disease

## Competing interests

The authors declare that they have no competing interests.

## Authors' contributions

BY was the main investigator and wrote the first draft. VT contributed to the study design and the data analysis. AFK contributed to the exercise therapy sessions and the programme management. AM contributed to the data analysis and wrote the final manuscript. All authors read and approved the paper.
